# Nature's Contributions to Human Health: A Missing Link to Primary Health Care? A Scoping Review of International Overview Reports and Scientific Evidence

**DOI:** 10.3389/fpubh.2020.00052

**Published:** 2020-03-18

**Authors:** Laura Lauwers, Hilde Bastiaens, Roy Remmen, Hans Keune

**Affiliations:** ^1^Department for Interdisciplinary and Primary Care, University of Antwerp, Wilrijk, Belgium; ^2^Research Institute Nature & Forest (INBO), Belgian Biodiversity Platform, Brussels, Belgium

**Keywords:** primary health care, nature, health, infectious diseases, natural disasters, medicinal plants, nature-based care

## Abstract

Nature's contributions to human health (NCH) have gained increased attention internationally in scientific and policy arenas. However, little attention is given to the role of the health care sector in this discussion. Primary health care (PHC) is a vital backbone for linking knowledge and practice within the organization of health care. The objective of this scoping review is to evaluate how international overview reports and scientific literature on NCH address to PHC. More specifically, we extracted data on arguments, practice supporting tools and guidelines, challenges and constraints, and management approaches to integrate NCH and PHC. The scientific literature search was run in Web of Science. Two independent reviewers screened the scientific publications. Through the scientific literature search, we identified 1,995 articles of which 79 were eligible for analysis. We complemented the search with a selection of six international overview reports. Both the international overview reports and the scientific publications paid limited attention to the role of PHC regarding NCH. To cope with the current challenges and constraints to integrate NCH and PHC, more evidence on NCH, further development of PHC practice supporting tools, bottom–up integrated approaches, and closer interdisciplinary collaborations are required.

## Introduction

Nature is known to affect human health in different ways. The manner in which nature's contributions to human health (NCH) have been studied reflects the changes in concepts of health. Health has long been defined as “the absence of disease” and approached by focusing on the pathogenesis or mechanisms that cause diseases (1). In this context, nature is simultaneously considered a threat to health due to the cause of diseases associated with mass mortalities and a source for curative care through the provision of medicinal compounds. In 1978, the World Health Organization (WHO) redefined the concept of health as “a state of complete physical, mental and social well-being and not merely the absence of disease or infirmity” in the Alma-Ata Declaration (1). This change of health concept illustrates the shift in mainly focusing on disease-causing factors to considering factors supporting human health and well-being. In this context, only recently, more attention is paid to the benefits of nature contact to human health. The range of mechanisms, from supporting physical activity to enhancing immune function, and effects of nature contact to human health, from improved mental health to reduced diabetes, were recently reviewed (2).

This shift from emphasizing health risks to including health benefits from nature is reflected in international overview reports of interdisciplinary collaborations on NCH. In 2005, the WHO contributed to the Millennium Ecosystem Assessment (MEA) with a state-of-the-art overview of the broad range of NCH (3). In this overview, the relation between human health and an ecosystem approach of nature is addressed. The review focuses on the risks of the degradation of ecosystems to human health, such as natural disasters and malnutrition. In 2010, the United Nations Food and Agriculture Organization (FAO), the World Organization for Animal Health (OIE), and the WHO produced a collaborative statement on One Health (4). This collaboration aimed at sharing responsibilities and coordinating global activities to address health risks at the animal–human–ecosystems interfaces (4). In parallel, Wildlife Trust and the Consortium for Conservation Medicine joined the EcoHealth Alliance, an international nonprofit organization dedicated to a “One Health” approach to protecting the health of people, animals, and the environment from emerging infectious diseases (5). In 2015, the initial steps in the collaboration between WHO and the Convention on Biological Diversity (CBD) regarding biodiversity and health began (6). The state of knowledge review resulting from this collaboration includes health benefits of nature in terms of biodiversity. In this period, the Rockefeller Foundation–Lancet Commission on planetary health introduces the concept of planetary health as an alternative to One Health and EcoHealth (5). The commission defined this planetary health as “the achievement of the highest attainable standard of health, well-being, and equity worldwide through judicious attention to the human systems—political, economic, and social—that shape the future of humanity and the Earth's natural systems that define the safe environmental limits within which humanity can flourish” (5). In 2016, the European OneHealth/EcoHealth (OH/EH) workshop aimed at integrating these concepts given the similarities in their objectives (7). Here again, the benefits of nature to human health are included in the final report. In the same period, the European Union itself published a recent report on nature-based solutions (NBS) and renaturing cities from a European expert group commissioned by the Directorate-General for Research and Innovation (8). In this report, the potential human health benefits of NBS are explicitly addressed, as are in research calls related to that report. However, despite growing interest from scientific and policy arenas for NCH, the role of the health care sector, and more specifically the role of PHC in relation to NCH, has not been thoroughly reviewed.

The integration of NCH and PHC fits well in the initial WHO 1978 vision of PHC as to be comprehensive (1). Comprehensive PHC encourages to improve social and environmental contexts that create disease and risks for disease and pays attention to disease prevention and health promotion (9). Due to pragmatic reasons, a more selective approach with marginalized attention for preventive and promotive actions took over this comprehensive vision, especially in low- and mid-income countries (9). However, today, there is an increased demand to close the gap between PHC and Public Health by strengthening the preventive and promotive character of PHC (10). The role of PHC has become strategically important for several reasons. PHC is the first level of contact of individuals, the family, and community with the national health system (1). Its coordinating role helps people to navigate the maze of health services and to mobilize the support of other facilities by referring patients or calling on the support of specialized services (11). A further asset is the close link between health care practice and local communities, which potentially makes it an ideal sector for implementing and communicating scientific findings of NCH research (1). Additionally, PHC plays an important role in coaching and medical follow-up for patients with chronic diseases, and therefore lowering the threshold for nature-based interventions among that vulnerable group. Thus, it is important to look at NCH in relation to PHC, and as a first step, we examined what has already been published. The objective of this scoping review is to evaluate how international overview reports and scientific literature on NCH address to PHC. To scope the available knowledge, the following research question was developed: What does the literature mention on the integration of NCH and PHC? To do so, this scoping review summarizes the literature into four themes: arguments, practice supporting tools and guidelines, challenges and constraints, and management approaches characterizing the integration of NCH and PHC.

## Methods

### Definitions

*Nature-based care:* we developed this umbrella term for health care interventions related to nature, such as green prescriptions, nature-based health interventions, nature-assisted therapies, and green care.

*Green Prescription:* a physical activity scripting scheme (written and verbal) whereby patients are initially screened for physical inactivity and receive a physical activity prescription from their GP (12).

*Nature-based health intervention:* a program, activity, or strategy that aims to engage people in nature-based experiences with the specific goal of achieving improved health and well-being (13).

*Nature-assisted therapy*: an intervention with the aim to treat, hasten recovery, and/or rehabilitate patients with a disease or a condition of ill health, with the fundamental principle that the therapy involves plants, natural materials, and/or outdoor environment, without any therapeutic involvement of extra human mammals or other living creatures (14, 15).

*Green care:* another umbrella term for denoting interventions that use elements of nature and defined as a targeted therapeutic or treatment intervention that is specifically designed for people with a defined need and is delivered by trained/qualified practitioners. However, this term has very broad implications, also including social rehabilitation or health promotion, and also contains animal-assisted therapy (14, 15).

*Complementary medicine or alternative medicine:* a broad set of health care practices that are not part of a country's own tradition or conventional medicine and are not fully integrated into the dominant health-care system (16).

### Protocol

A full review protocol was drafted *a priori* by the research team (LL, HK, RR, and HB), and is available in JMIR protocols (https://www.researchprotocols.org/2019/1/e12510/) (17). A summary of the methodology follows.

### Search Criteria

We used a two-step approach to explore the domain of NCH in relation to PHC. First, we searched the gray literature for recent international overview reports on NCH. We complemented this by a scientific literature search in Web of Science (WOS). With the help of PHC professionals and the application of PubMed Search Builder, we developed a search string for PHC. This search string was combined with search strings for nature and the nature–health subthemes presented in the WHO-CBD report: human microbiome, infectious diseases, natural disasters, medicinal plants, and nutrition (6). These search strings were adopted from the search strategy for the Regional Assessment for Europa and Central Asia by the Intergovernmental Platform on Biodiversity and Ecosystem Services (IPBES) (18). We developed an additional subtheme “nature-based care” and considered it as an umbrella term for health care interventions related to nature. The diverse range of topics covered by those nature–health subthemes allowed to fully grasp the full potential connections between all aspects of NCH and PHC. The relevance of the subthemes and an overview of the search strings are presented in the published protocol (17). The search strings were combined as follows:

- Nature–health subtheme AND primary health care.
- Nature–health subtheme AND primary health care AND nature.
- Nature AND primary health care.

### Inclusions and Exclusion Criteria

**Figure 1:**
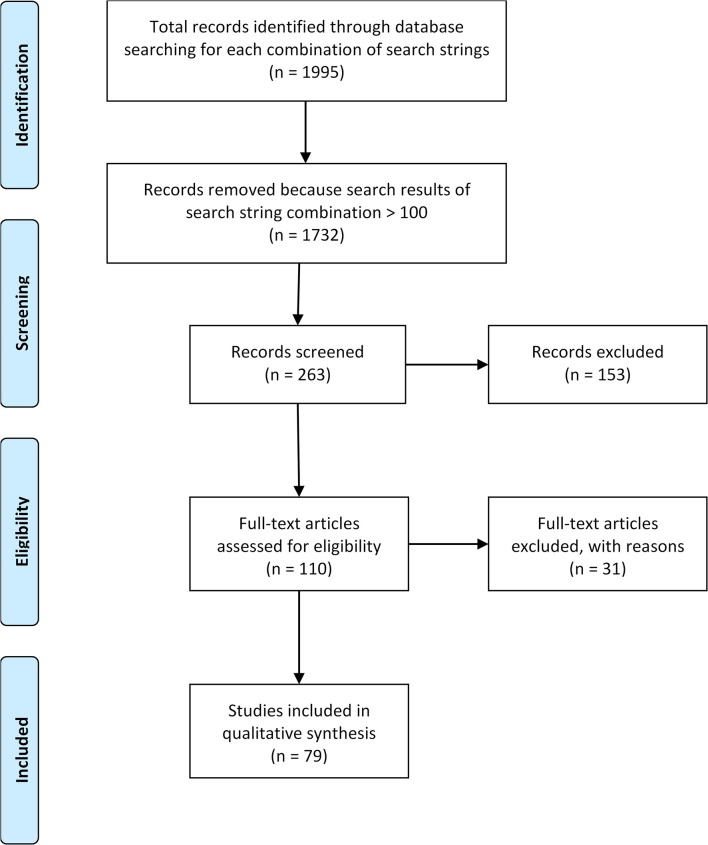
Prisma diagram of articles included in study.

We considered all literature dating from 1900 to May 2017. We did not consider foreign language material, except for papers with an English abstract because of the cost and time involved for translation. Two independent reviewers (first reviewer: HK, second reviewer: HB or RR) checked the titles and abstracts of the search results for relevance. Relevance was attributed if the publications approached the nature–health subthemes in accordance to their relevance to health and if they paid attention to PHC in a nonsuperficial manner. Publications were included when explicitly relating the research findings on NCH to PHC, with references to PHC according to the keywords of the search string. Publications were excluded from further analysis when only mentioning PHC, but not linking the NCH knowledge to PHC. At title and abstract stage, for each publication, reviewers made one of following decisions: relevant, in doubt, or irrelevant. In the case of doubt or disagreement, the first reviewer made a final decision at the full paper stage in consultation with the second reviewer. As we were more interested to explore a broad range of topics related to NCH than to go into great detail on a specific topic, for pragmatic reasons, papers were only checked for relevance when the number of results of a combination of search strings was below 100. Reviews were always considered. The quality of the papers was not assessed. Figure 1 illustrates the selection process for the scientific literature. More details can be found in the published protocol (17).

### Extraction of Data

For the international overview reports, we only screened and extracted data with keywords associated to PHC. Since the results were very limited, we screened and extracted additionally data containing the keywords “health care,” “healthcare,” and “health prof.”

For the scientific literature, we read the full article and extracted data following a list of extraction fields, including “year of publication,” “country of origin,” and key findings that relate to the research question: What does the literature mention on the integration of NCH and PHC?

Based on first insights in the data extracted in relation to our research question, we decided during the development of our protocol to sort all data under four main categories: (1) arguments, (2) practice supporting tools and guidelines, (3) challenges and constraints, and (4) management approaches characterizing the integration of NHI in PHC. This data extraction approach is akin to a “narrative review,” where we used the four categories as an analytical framework to all data extracted from the international overview reports and scientific literature (19). We sought to apply a uniform approach to all literature included in the review, but in practice it was sometimes impossible to extract information on all four categories where some literature failed to include relevant material. Since the spatial and temporal information of the scientific literature did not show remarkable trends in topical foci, we did not report on those results.

## Results

### Results From International Overview Reports

Six international overview reports on NCH were selected to complement the scientific literature (Table 1).

**Table 1:** Selected international overview reports.

**Report reference**	**Title of report**	**Additional information**
WHO (3)	Ecosystems and Human Well-Being: Health Synthesis	Report of the Millennium Ecosystem Assessment
Whitmee et al. (20)	Safeguarding human health in the Anthropocene epoch	Report of The Rockefeller Foundation-Lancet Commission on planetary health
WHO-CBD (6)	Connecting global priorities: biodiversity and human health	State of knowledge review
Ten Brink et al. (21)	The Health and Social Benefits of Nature and Biodiversity Protection	Report for the European Commission—DG Environment
UNEP (22)	Healthy Environment, Healthy People.	Thematic report—Ministerial policy review
WHO (23)	Urban green spaces and health. A review of evidence	WHO Regional Office for Europe

The role of PHC remained mainly underreported in the selected international overview reports on NCH. The WHO contribution to the Millennium Ecosystem Assessment (3) and the UNEP report “Healthy Environment, Healthy People” (22) did not include key terms associated to PHC. Especially in the WHO–CBD review (6), attention to PHC became more specific. None of the reports referred to practice supporting tools or guidelines. Arguments, challenges and constraints, and management approaches to integrate NCH and PHC could be extracted from the reports (Supplementary Table 1).

#### Arguments

We derived several arguments from the reports to better integrate NCH and PHC. First of all, natural disasters already have a great impact on the (primary) health care costs and infrastructure (3, 6, 20). Contrary, (primary) health care itself has an impact on natural ecosystems that subsequently can have a negative effect on human health (6). Second, through the central role of PHC in communities, PHC possesses interesting health data that can be spatially linked to environmental data and strengthen the response and preparedness to health impacts of the environment (20, 23). This central role can further support health professionals to mobilize a wide community of actors to increase awareness for NCH (20, 21). Third, worldwide medicinal plant use continues to be an important part of (primary) health care systems where training local communities could help to protect biodiversity and local knowledge and to reduce poverty (3, 6). At last, evidence for the effectiveness and preventive potential of NCH is increasing and could reduce health care costs (21–23).

#### Challenges and Constraints

A big challenge is that poor populations are often more dependent on natural resources in their environment due to inadequate access to health care, but often live in areas that are becoming more prone to flooding or other natural disasters (20). Additionally, a minimum threshold of health-worker capacity is required to prevent and respond to the health consequences of environmental change (20). Besides the increase in health inequalities, the integration of NCH and PHC is constraint by the general trend of budget cuts in preventive care (21). This trend reflects the current challenge to increase awareness on the preventive functions of nature. Consequently, dedicated measures for awareness raising are required to facilitate the mainstreaming of NCH (21).

#### Management Approaches

From the extracted management approaches, we learn that an integration of PHC and NCH is required on different levels, involving different actors. First, knowing the reciprocal impact of the health care sector and the natural environment, the integration of NCH and PHC requires the integration of policies advancing both human health and environmental sustainability (20). Second, data integration should be obtained, for example, by including environmental metrics in facility and population-based health surveys (20). Third, interdisciplinary collaborations between health and environmental actors are required and could be stimulated by the local authorities through collaboration calls or collaboration platforms (20–22). Fourth, the integration of NCH and PHC can be further enabled by providing trainings: trainings to local community members to protect their natural resources, to researchers to gain skills in producing and commercializing traditional medicines, and to health professionals in understanding and applying concepts and practices of NCH (6, 20, 21). Regarding medicinal plant research, the reports emphasize the importance of respecting local health culture and knowledge, for example, by informing the local community on the research project or by developing memoranda of understanding between researchers and traditional healers (6, 20). Fifth, NCH and PHC could be spatially integrated by establishing medicinal plant conservation areas, planning green space for health, recognizing nature areas as “preventive health care centers” or “health hubs,” financially supporting health care services to utilize urban green spaces, or incorporating green areas in health services. Health professionals can take an active role in this integration by joining environmental health research, writing green referrals, and using their central role to mobilize a wide community of actors (6, 20, 21). At last, the integration can be stimulated by copying good practices on small scales and applying them on a wider set of regions (6, 21).

### Results From Scientific Literature

We identified 1,995 articles of which 79 (28 reviews, 51 papers) were eligible for analysis. Table 2 gives a quantitative overview of the search string combinations. The last column illustrates the total amount of reviews and papers included for a specific nature–health subtheme. Some articles were moved to the results of another subtheme if they fitted better content-wise. We did not identify relevant articles that fitted the nature–health subthemes “human microbiome” and “nutrition.”

**Table 2:** Quantitative overview of the search string combinations applied in WOS.

**Nature–health subthemes**	**Reviews**		**Papers**		**Total checked**	**Total included**
	**PHC^a^**	**PHC + nature**	**PHC**	**PHC + nature**		
Nature	33	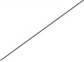	424^b^	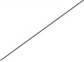	33	1
Nature-based care	3	0	35	2	40	32
Human microbiome	4	1	14	1	20	0
Nutrition	1	0	16	1	18	0
Medicinal plants	22	2	198^b^	35	59	31
Infectious diseases	14	1	202^b^	5	20	12
Natural disasters	72	0	901^b^	1	73	3

a *PHC, primary health care*.

b *Articles that were not considered since the search resulted in a total amount higher than 100*.

The results of each search string combination with PHC that were considered relevant to our research question showed considerable differences in topical foci (Supplementary Table 2). The scientific literature allowed us to draw lessons on arguments, practice supporting tools and methods, challenges, constraints, and management approaches to integrate NCH and PHC.

#### Arguments

The arguments to integrate NCH and PHC relate strongly to the comprehensive vision of PHC by focusing on the potential for health promotion and disease prevention, both on individual and population level. Literature regarding “nature-based care” describes the potential of PHC in disease prevention on the individual level through health-promoting interventions, e.g., physical activity interventions, nature-based care, and complementary therapies. On a population level, this literature emphasizes the potential of PHC professionals to have a broad public health impact as they are considered highly credible resources for health information and are often visited on a regular basis by their patients (12, 24). Literature regarding “medicinal plants” describes the potential of PHC in disease prevention on the individual level through holistic care interventions, for example, Ayurveda complementary therapies. On a population level, this literature emphasizes that although interventions of conventional medicines dominate in the western countries, three quarters of the world population rely on herbal and traditional medicine as a basis for PHC because of its affordability, accessibility, and long cultural history (25–31). As traditional medicinal knowledge is disappearing, PHC has the potential to conserve and integrate this knowledge as a response to the growing demand for traditional healing, also in western countries (28, 32). Further, literature regarding both “nature-based care” and “medicinal plants” underlines a reduction in consumption and costs of PHC as a result of the preventive and therapeutic potential of the presented interventions (29, 33–37). Literature regarding “infectious diseases” and “natural disasters” describes the potential of PHC in disease control and prevention only on the population level through early interventions during the detection and treatment of diseases (38–40).

#### Practice Supporting Tools and Guidelines

Only two articles of the included literature provide tools to support PHC workers that directly relate to NCH. One article within the subtheme “infectious diseases” introduces a pathogen source questionnaire to gain insights in the zoonotic contact of the patient (41). The other article within “natural disasters” considers two summarized checklists, one for the early warning system, as well as when doing post-disaster risk assessment, and the other for prevention and control of recorded diseases (38). Two articles within “natural disasters” provide useful traumatic screening instruments, but mentioned “natural disasters” only once as an example of possible trauma exposures (42, 43). Although not published as a guideline, one article on “natural disasters” reviews all skin diseases related to floods and summarized the appropriate management or treatment (39). “Nature-based care” literature discusses specific trainings to support the PHC professional in writing green prescriptions (34, 44–46). To give written advice to a patient to be physically active, PHC trainings on motivational interviewing techniques and behavioral counseling strategies are recommended (34, 45, 47). However, again these articles lack references to specific practice supporting guidelines and refer mostly to physical activity in general without giving attention to the natural component (12, 24).

#### Challenges and Constraints

Literature concerning “nature-based care” and “medicinal plants” assigns the difficulty to integrate NCH and PHC to a general lack of collaboration and communication between different areas of knowledge (48–50). Where ethnobotanical research is predominated by descriptive works of useful plants, the translation of this traditional knowledge into PHC practice requires more interchange of theories and methods among related disciplines, like ethnopharmacology (48). Furthermore, the development of wide-scoped interdisciplinary projects that recognize intellectual property rights and reward the communities for their knowledge contribution are needed to counteract the disappearance of traditional medicinal knowledge, opposing the growing demand for traditional healing (48, 49). Articles about “nature-based care” provide several reasons to explain why the green prescribing practice remains low despite the growing evidence on the health benefits of green space (50). First of all, this interdisciplinary field requires the development of a consistent terminology to support a better collaboration. Besides a need for interdisciplinary collaboration, a behavioral change is required both from the patient and the PHC professional as the new approaches extend the traditional biomedical model (24, 34, 45, 46). However, the lack of resources and time in the PHC sector greatly challenge the practical trainings and substantial support from the physician toward the patient required for these behavioral changes to succeed (24, 34, 46). At last, where strong evidence is needed to convince PHC professionals of a certain practice, there is little academic interest in the field, for example, for phytotherapy, or the type of evidence cannot compete with evidence base using methodologies like randomized controlled trials (RCTs) (36, 50). The type of evidence used to indicate the effects of an intervention is mostly based on subjective measures, like self-reported health status (51–53). The latter is also reflected in the literature on “medicinal plants,” where the evidence for the healing effects of medicinal plants is often limited to the traditional, and, to a certain extent, subjective use of plants by local communities (26).

#### Management Approaches

The paragraphs below illustrate that the integration of NCH and PHC should preferably be done in a bottom–up manner and that horizontal networks among PHC practices and community-based facilities should be stimulated. Articles addressing “medicinal plants” emphasize the importance of community participation in studies on traditional knowledge acquisition. These articles tell us that local communities and local stakeholders are willing to contribute to scientific research and participate in the conservation of medicinal plants and the associated knowledge, but that the state health care negatively influences traditional-based healing practices by promoting allopathic health care (28, 49, 54). Literature regarding "infectious diseases” discusses community participation in relation to successful disease control (55–59). Decentralized or inadequate public health infrastructure, shortage of financial resources, and human resistance to programs emerge as reasons for failures of vertical health projects and a shift to community-oriented PHC systems in many countries (55–59). To engage community members in public health programs, community participants should define their idea of “participation” and what they perceive as their “communities” (36). For “nature-based care,” literature discusses community involvement in PHC mainly in relation to physical activity programs (24, 34, 45, 46, 58, 59). These studies show that giving advice only to increase physical activity through green prescriptions is not as effective as tailored interventions involving a personalized action plan supported by a strong network between PHC and sport or other facilities in the community (24, 46, 58). Such a network builds the opportunity to share the load of intervention activities, as PHC professionals are limited by consultation time (24, 34). These communal facilities can help in motivating and monitoring the patients in the performance of their physical activities or in providing opportunities for health promotion in general, e.g., the provision of community vegetable gardens or health education activities (24, 34, 45, 46, 58–61).

## Discussion

This review aimed to get an overview of the state of the art of NCH in relation to PHC. To this end, this review indicates that a potential role for PHC in NCH is perhaps not fully recognized. The role of PHC was most clearly defined in the literature on “nature-based care” in terms of disease prevention and health promotion through the application of green prescriptions, and in the literature on “medicinal plants” in terms of curative care through the application of knowledge on traditional medicine. Additionally, the international overview reports highlighted the potential role of PHC professionals in mobilizing a wide community and contributing to environmental health research. However, the included literature did not cover specific tools to support PHC practices. PHC practice- supporting tools and guidelines were in general limited and very context-specific. The given tools and guidelines were based on an overall recognition of the importance of “context” to integrate NCH and PHC. Not only increased attention to the patient's history and background but also to the context of the PHC professional and of the health issue itself was mentioned by the included literature. The limited number of tools and guidelines presented in the included literature might on one hand be associated with the underrecognition of evidence on NCH by health professionals, since the available data deviates from the strict criteria for RCTs (50). On the other hand, some research fields within NHC are quite recent, such as the contribution of microbial diversity in the natural environment to immune function. Despite a lack of practice supporting tools and guidelines, the review covered a wide range of arguments to integrate NCH and PHC. Most arguments related to the economic costs of PHC, the preventive potential of NCH, and the need to protect our natural health resources. However, this integration is not without challenges and constraints. The included literature highlighted the need to tackle health inequalities related to poverty, to better inform on NCH among PHC professionals, to improve interdisciplinary collaborations, and to provide PHC practices with the necessary resources. Finally, the scientific literature and international overview reports emphasized the need for integrated, bottom–up approaches to successfully link NCH and PHC. Regarding these management approaches, the included literature highlighted the importance of respecting local environmental and health knowledge, linking environmental and health data collection, and a better coordination of environmental and health policies. However, at present, policy makers seem to struggle to scale up these integration models, while at the same time responding to local conditions and needs of the community (62).

The findings of this review should be viewed in light of limitations in our method. The main limitation of the review is the lack of capacity for a content analysis when the number of the results crossed the self-determined threshold of 100. This implies that the included literature does not fully cover the quantitative presence of certain topics in the scientific literature.

By linking to PHC, this review adds priorities to the research agenda published by Frumkin et al. to guide future studies on NCH (63). Overall, knowledge on NCH in PHC and the role for PHC regarding the application of that knowledge in health care practice remains in its infancy in both science and practice. Based on this research, we suggest that research priority should be given to real-time examples of practice with the assessment of the process and time- and cost-effectiveness to identify best practices. Based upon this research, necessary tools and guidelines to support the integration of NCH and PHC could be developed. The growing interest for green prescriptions opens research opportunities to investigate benefits of nature on health in a PHC setting. As the included literature is mostly restricted to physical activity in general without mentioning the benefits related to physical activity in a natural setting, for example, the improvement in mental well-being or immune function, it would be interesting to further investigate the added value of a natural environment for physical activities and how the patient can be stimulated through a community-based referral network to visit natural environments for physical activities (64–66). Regarding medicinal plants, this review provides an overview of scientific literature that can serve as a starting point to develop management plans urgently needed to conserve the medicinal resources and the associated traditional knowledge. The literature on infectious diseases relates to the One Health approach by emphasizing the necessity to further investigate ways to strengthen the collaboration between environmental, human, and animal health care sectors for disease control and prevention, with PHC being an ideal setting to converge those disciplines (20). The unpredictable outcomes of natural disasters might challenge the reporting of NCH in the context of such events; however, with the growing climate threat, these reports become highly important to mobilize PHC in an efficient manner.

PHC provides a relevant and valuable evaluation testing ground for studies on the integration of NCH in health care practices. PHC can report back to research, as it is close to community health developments, and as such delivers important data to science and policy. Further, it focuses on reducing health inequalities in communities (67). To conclude, this review provides a broad overview of the potential of integrating NCH and PHC and calls for a better uptake of this potential in future scientific studies and international policy directives on NCH.

## Data Availability Statement

All data generated or analyzed during this study are included in this published article and in the published protocol of this article (17).

## Author Contributions

HK, RR, and HB conceptualized the review and involved in the selection of relevant scientific literature. HK developed the search strategy with guidance from Conor Kretsch (COHAB) and Bram Oosterbroek (ICIS-University of Maastricht). LL analyzed the literature and wrote the manuscript of the review with critical inputs and appraisal from HK, RR, and HB. All authors have read and approved the manuscript.

### Conflict of Interest

The authors declare that the research was conducted in the absence of any commercial or financial relationships that could be construed as a potential conflict of interest.
