# Supplementary material for: Nature's Contributions to Human Health: A Missing Link to Primary Health Care? A Scoping Review of International Overview Reports and Scientific Evidence
Source: Front Public Health. 2020 Mar 18;8:52. doi: 10.3389/fpubh.2020.00052 (PMC7093563; doi:10.3389/fpubh.2020.00052)
Supplement: Supplementary file 1 [file Table_1.docx]

Supplementary Material

Table 1 Thematic description of the international overview reports.

| **Themes associated to primary health care, health professionals and health care in general** | WHO (2005) [3] | Whitmee et al. (2015) [21] | WHO-CBD (2015) [6] | Ten Brink et al. (2016) [23] | UNEP (2016) [20] | WHO (2016) [22] |
| --- | --- | --- | --- | --- | --- | --- |
| ARGUMENTS | | | | | | |
| Economic cost | + |  | + | + | + | + |
| Centrality |  | + |  | + |  | + |
| Health - environment impact |  |  | + |  |  |  |
| Medicinal plants | + |  | + |  |  |  |
| Effectiveness of NBI^a^ |  |  |  | + |  |  |
| Disease prevention |  |  |  | + | + | + |
| CHALLENGES & CONSTRAINTS | | | | | | |
| Poverty |  | + | + |  |  |  |
| Budget cuts in preventive care |  |  |  | + |  |  |
| Need for increased awareness |  |  |  | + |  |  |
| Health-worker capacity |  | + |  |  |  |  |
| Awareness raising measures |  |  |  | + |  |  |
| MANAGEMENT APPROACHES | | | | | | |
| Policy integration |  | + |  |  |  |  |
| Interdisciplinary collaboration |  | + |  | + | + |  |
| Data integration |  | + |  | + |  | + |
| Informing and training |  | + | *+* | *+* |  |  |
| Local knowledge and culture |  | + | + |  |  |  |
| Spatial integration |  |  | + | + |  |  |
| Role of health professionals |  | + | + | + |  |  |
| Role of local authorities |  |  |  | + |  |  |
| Copy local initiatives |  |  | + | + |  |  |

^a^NBI: nature-based interventions.
