# Supplementary material for: Nature's Contributions to Human Health: A Missing Link to Primary Health Care? A Scoping Review of International Overview Reports and Scientific Evidence
Source: Front Public Health. 2020 Mar 18;8:52. doi: 10.3389/fpubh.2020.00052 (PMC7093563; doi:10.3389/fpubh.2020.00052)
Supplement: Supplementary file 2 [file Table_2.docx]

Supplementary Material

Table 2 Topical description of included scientific literature on the different nature-health subthemes.

| **Nature-health subtheme** | **Key topic** | **Review** | **Paper** |
| --- | --- | --- | --- |
| Nature | Probiotics | Dai et al (2013)[68] |  |
| Nature-based care | Physical activity | Arsenijevic & Groot (2017)[53]; Estabrooks & Glasgow (2006)[24]; Huijg et al (2014)[46] | Anderson et al (2015)[47]; Croteau et al (2006)[12]; Dalziel et al (2006)[33]; Elley et al (2003)[45]; Elley et al (2011)[69]; Gribben et al (2000)[70]; Hamlin et al (2016)[59]; Huijg et al (2014)[71]; Kerse et al (2005)[72]; Kolt et al (2009)[73]; Kolt et al (2012)[74]; Korpela et al (2008)[51]; Lamb et al (2002)[58]; Leung et al (2012)[75]; Lobelo et al (2014)[34]; Patel et al (2011)[76]; Patel et al (2013)[77]; Patel et al (2013)[78]; Swinburn et al (1997)[44]; Swinburn et al (1998)[79]; Van den Berg (2017)[50]; Wannamethee et al (2000)[80] |
|  | Nature-assisted therapy |  | Barley et al (2012)[52]; Fromageot et al (2005)[81]; Wahrborg et al (2014)[35] |
|  | Urban nature | Costa et al (2015)[60]; Pereira et al (2009)[61] | Nayyar & Hwang (2015)[82] |
|  | Medicinal plants |  | Balick (1990)[83] |
| Medicinal plants | Complementary therapies | Antonio et al (2014)[36]; Dikshit, et al (2004)[84]; Schveitzer & Zoboli (2014)[32] | Dal Cero et al (2015)[85] |
|  | Traditional use & knowledge | Grierson et al (2014)[86]; Hossen et al (2016)[37]; Maroyi (2013)[27]; Mishra et al (2016)[87]; Sharma et al (2007)[88] | Ahmad et al (2015)[89]; Aziz et al (2016)[90]; Baydoun et al (2015)[91]; Bermúdez et al (2005)[48]; Bhattarai et al (2010)[49]; Da Silva et al (2012)[92]; de Boer et al (2012)[26]; Idu et al (2007)[93]; Ikram et al (2015)[94]; Jain et al (2005)[95]; Malla et al (2015)[96]; Mathibela et al (2015)[28]; Mootoosamy & Mahomoodally (2014)[97]; Panyaphu et al (2011)[54]; Rasool et al (2016)[29]; Semenya et al (2012)[98]; Semenya et al (2013)[99]; Shil et al (2014)[30]; Soejarto et al (2012)[100]; Vandebroek et al (2004)[31] |
|  | Toxicities | Efferth & Kaina (2011)[25] |  |
|  | Anti-infective drugs | Husain and Ahmad (2013)[101] |  |
| Infectious diseases | Hand-washing techniques | Smith (2009)[102] |  |
|  | Enteric protozoa | Fletcher et al (2012)[103] |  |
|  | Zoonotic diseases | Beeckman & Vanrompay (2009)[104] | Day (2016)[105]; Hensgens et al (2012)[106]; Warwick (2004)[41] |
|  | Disease control | Atkinson et al (2011)[56]; Corley et al (2016)[57]; Lines et al (1994)[55]; Haider et al (2015)[40] |  |
|  | Natural disasters | Kouadio et al (2012)[38] |  |
|  | Soil-Related Infections | Baumgardner (2012)[107] |  |
| Natural disasters | Traumatic experiences | Crosby (2013)[42], Spoont et al (2015)[43] |  |
|  | Flood-related skin diseases | Tempark et al (2013)[39] |  |

References

68. Dai C, Zheng CQ, Jiang M, Ma XY, Jiang LJ. Probiotics and irritable bowel syndrome. World journal of gastroenterology (2013) 19:5973–80. doi: 10.3748/wjg.v19.i36.5973

69. Elley CR, Garrett S, Rose SB, O'Dea D, Lawton BA, Moyes SA, et al. Cost-effectiveness of exercise on prescription with telephone support among women in general practice over 2 years. Br J Sports Med (2011) 45:1223–9. doi: 10.1136/bjsm.2010.072439

70. Gribben B, Goodyear-Smith F, Grobbelaar M, O'Neill D, Walker S. The early experience of general practitioners using Green Prescription. N Z Med J (2000) 113:372–3.

71. Huijg JM, van der Zouwe N, Crone MR, Verheijden MW, Middelkoop BJ, Gebhardt WA. Factors influencing the introduction of physical activity interventions in primary health care: a qualitative study. Int J Behav Med (2014) 22:404–14. doi: 10.1007/s12529-014-9411-9

72. Kerse N, Elley CR, Robinson E, Arroll B. Is physical activity counseling effective for older people? A cluster randomized, controlled trial in primary care. J Am Geriatr Soc (2005) 53:1951–6. doi: 10.1111/j.1532-5415.2005.00466.x

73. Kolt GS, Schofield GM, Kerse N, Garrett N, Ashton T, Patel A. Healthy Steps trial: pedometer-based advice and physical activity for low-active older adults. Annals of family medicine (2012) 10:206–12. doi: 10.1370/afm.1345

74. Kolt GS, Schofield GM, Kerse N, Garrett N, Schluter PJ, Ashton T et al. The healthy steps study: a randomized controlled trial of a pedometer-based green prescription for older adults. Trial protocol. BMC Public Health (2009) 9:404. doi: 10.1186/1471-2458-9-404

75. Leung W, Ashton T, Kolt GS, Schofield GM, Garrett N, Kerse N, et al. Cost-effectiveness of pedometer-based versus time-based Green Prescriptions: the Healthy Steps Study. Australian journal of primary health (2012) 18:204–11. doi: 10.1071/PY11028

76. Patel A, Schofield GM, Kolt GS, Keogh JWL. General practitioners views and experiences of counselling for physical activity. BMC Family Practice (2011) 12:119–26. doi: 10.1186/1471-2296-12-119

77. Patel A, Keogh JW, Kolt GS, Schofield GM. The long-term effects of a primary care physical activity intervention on mental health in low-active, community-dwelling older adults. Aging & mental health (2013) 17:766–72. doi: 10.1080/13607863.2013.781118

78. Patel A, Schofield GM, Kolt GS, Keogh JW. Perceived barriers, benefits, and motives for physical activity: two primary-care physical activity prescription programs. Journal of aging and physical activity (2013) 21:85–99. doi: 10.1123/japa.21.1.85

79. Swinburn BA, Walter LG, Arroll B, Tilyard MW, Russell DG. The green prescription study: a randomized controlled trial of written exercise advice provided by general practitioners. Am J Public Health (1998) 88:288–91. doi: 10.2105/AJPH.88.2.288

80. Wannamethee SG, Shaper AG, Walker M. Physical activity and mortality in older men with diagnosed coronary heart disease. Circulation (2000) 102:1358–63. doi: 10.1161/01.CIR.102.12.1358

81. Fromageot A, Parent F, Coppieters Y. Femmes, cultures maraîchères et recours aux soins en Afrique de l'Ouest. Sciences Sociales et Santé (2005) 23:49–70.

82. Nayyar D, Hwang SW. Cardiovascular Health Issues in Inner City Populations. Can J Cardiol (2015) 31:1130–8. doi: 10.1016/j.cjca.2015.04.011

83. Balick MJ. Ethnobotany and the identification of therapeutic agents from the rainforest. Ciba Foundation symposium (1990) 154:22–31. doi: 10.1002/9780470514009.ch3

84. Dikshit A, Shahi SK, Pandey KP, Patra M, Shukla AC. Aromatic plants - a source of natural chemotherapeutants. National Academy Science Letters (2004) 27:145–64.

85. Dal Cero M, Saller R, Weckerle CS. Herbalists of Today's Switzerland and Their Plant Knowledge. A Preliminary Analysis from an Ethnobotanical Perspective. Forschende Komplementarmedizin (2015) 22:238–45. doi: 10.1159/000438809

86. Grierson D, Otang WM, Afolayan AJ. A review of the Botany, phytochemistry, pharmacology and toxicology of Arctotis arctotoides (L.f.) O.Hoffm. (Asteraceae). Afr J Tradit Complement Altern Med (2014) 11:118–26. doi: 10.21010/ajtcam.v11i6.12

87. Mishra A, Seth A, Maurya SK. Therapeutic significance and pharmacological activities of antidiarrheal medicinal plants mention in Ayurveda: A review. Journal of intercultural ethnopharmacology (2016) 5:290–307. doi: 10.5455/jice.20160426094553

88. Sharma H, Chandola HM, Singh G, Basisht G. Utilization of Ayurveda in health care: an approach for prevention, health promotion, and treatment of disease. Part 2-Ayurveda in primary health care. Journal of alternative and complementary medicine (2007) 13:1135–50. doi: 10.1089/acm.2007.7017-B

89. Ahmad M, Khan MP, Mukhtar A, Zafar M, Sultana S, Jahan S. Ethnopharmacological survey on medicinal plants used in herbal drinks among the traditional communities of Pakistan. J Ethnopharmacol (2016) 184:154–86. doi: 10.1016/j.jep.2016.02.039

90. Aziz MA, Adnan M, Khan AH, Rehman AU, Jan R, Khan J. Ethno-medicinal survey of important plants practiced by indigenous community at Ladha subdivision, South Waziristan agency, Pakistan. Journal of ethnobiology and ethnomedicine (2016) 12:53. doi: 10.1186/s13002-016-0126-7

91. Baydoun S, Chalak L, Dalleh H, Arnold N. Ethnopharmacological survey of medicinal plants used in traditional medicine by the communities of Mount Hermon, Lebanon. J Ethnopharmacol (2015) 173:139–56. doi: 10.1016/j.jep.2015.06.052

92. Da Silva NCB, Delfino Regis AC, Esquibel MA, Espirito Santo Santo J, Almeida MZ. Medicinal plants use in Barra II quilombola community - Bahia, Brazil. Boletín Latinoamericano y del Caribe de Plantas Medicinales y Aromáticas (2012) 11:435–453

93. Idu M, Osemwegie O, Odia EA, Onyibe HI. A survey of indigenous flora used by folk medicine practitioners in Yola council area of Adamawa State, Nigeria. Plant Archives (2007) 7:517–521.

94. Ikram A, Zahra NB, Shinwari ZK, Qaiser M. Ethnomedicinal review of folklore medicinal plants belonging to family Apiaceae of Pakistan. Pakistan Journal of Botany (2015) 47:1007–14.

95. Jain A, Katewa SS, Galav PK, Sharma P. Medicinal plant diversity of Sitamata wildlife sanctuary, Rajasthan, India. J Ethnopharmacol (2005) 102:143–57. doi: 10.1016/j.jep.2005.05.047

96. Malla B, Gauchan DP, Chhetri RB. An ethnobotanical study of medicinal plants used by ethnic people in Parbat district of western Nepal. J Ethnopharmacol (2015) 165:103–17. doi: 10.1016/j.jep.2014.12.057

97. Mootoosamy A, Fawzi Mahomoodally M. Ethnomedicinal application of native remedies used against diabetes and related complications in Mauritius. Journal of ethnopharmacology (2014) 151:413–44. doi: 10.1016/j.jep.2013.10.069

98. Semenya S, Potgieter M, Tshisikhawe M, Shava S, Maroyi A. Medicinal utilization of exotic plants by Bapedi traditional healers to treat human ailments in Limpopo province, South Africa. J Ethnopharmacoly (2012) 144:646–55. doi: 10.1016/j.jep.2012.10.005

99. Semenya SS, Potgieter MJ, Erasmus LJ. Exotic and indigenous problem plants species used, by the Bapedi, to treat sexually transmitted infections in Limpopo Province, South Africa. Afr Health Sci (2013) 13:320–6. doi: 10.4314/ahs.v13i2.17

100. Soejarto DD, Gyllenhaal C, Kadushin MR, Southavong B, Sydara K, Bouamanivong S et al. An ethnobotanical survey of medicinal plants of Laos toward the discovery of bioactive compounds as potential candidates for pharmaceutical development. Pharmaceutical biology (2012) 50:42–60. doi: 10.3109/13880209.2011.619700

101. Husain F, Ahmad I. Quorum sensing iNCHbitors from natural products as potential novel anti-infective agents. Drugs of the Future (2013) 38:691–705. doi: 10.1358/dof.2013.038.10.2025393

102. Smith SM. A review of hand-washing techniques in primary care and community settings. Journal of clinical nursing (2009) 18:786–90. doi: 10.1111/j.1365-2702.2008.02546.x

103. Fletcher SM, Stark D, Harkness J, Ellis J. Enteric protozoa in the developed world: a public health perspective. Clin Microbiol Rev (2012) 25:420–49. doi: 10.1128/CMR.05038-11

104. Beeckman DS, Vanrompay DC. Zoonotic Chlamydophila psittaci infections from a clinical perspective. Clinical microbiology and infection (2009) 15:11–7. doi: 10.1111/j.1469-0691.2008.02669.x

105. Day MJ. Pet-Related Infections. Am Fam Physician (2016) 94:794–802.

106. Hensgens MP, Keessen EC, Squire MM, Riley TV, Koene MG, de Boer E, et al. Clostridium difficile infection in the community: a zoonotic disease? Clinical microbiology and infection (2012) 18:635-645. doi: 10.1111/j.1469-0691.2012.03853.x

107. Baumgardner DJ. Soil-related bacterial and fungal infections. JABFM (2012) 25:734–44. doi: 10.3122/jabfm.2012.05.110226
